# Impact of One-Carbon Metabolism-Driving Epitranscriptome as a Therapeutic Target for Gastrointestinal Cancer

**DOI:** 10.3390/ijms22147278

**Published:** 2021-07-06

**Authors:** Yu Takeda, Ryota Chijimatsu, Andrea Vecchione, Takahiro Arai, Toru Kitagawa, Ken Ofusa, Masami Yabumoto, Takaaki Hirotsu, Hidetoshi Eguchi, Yuichiro Doki, Hideshi Ishii

**Affiliations:** 1 Center of Medical Innovation and Translational Research, Department of Medical Data Science, Osaka University Graduate School of Medicine, Suita, Yamadaoka 2-2, Osaka 565-0871, Japan; takey8729@gmail.com (Y.T.); rchijimatsu@cfs.med.osaka-u.ac.jp (R.C.); t.arai@unitech-op.com (T.A.); toru@kyowakai.com (T.K.); oof21443@ideacon.co.jp (K.O.); yabumoto.masami@gmail.com (M.Y.); hirotsu@hbio.jp (T.H.); 2 Department of Gastroenterological Surgery, Graduate School of Medicine, Osaka University, Suita 565-0871, Japan; heguchi@gesurg.med.osaka-u.ac.jp (H.E.); ydoki@gesurg.med.osaka-u.ac.jp (Y.D.); 3 Department of Clinical and Molecular Medicine, University of Rome “Sapienza”, Santo Andrea Hospital, Via di Grottarossa, 1035-00189 Rome, Italy; andrea.vecchione@uniroma1.it; 4 Unitech Co., Ltd., Kashiwa 277-0005, Japan; 5 Kyowa-kai Medical Corporation, Osaka 540-0008, Japan; 6 Food and Life-Science Laboratory, Prophoenix Division, Idea Consultants, Inc., Osaka 559-8519, Japan; 7 Kinshu-kai Medical Corporation, Osaka 558-0041, Japan; 8 Hirotsu Bio Science Inc., Tokyo 107-0062, Japan

**Keywords:** one-carbon metabolism, amino acids, epitranscriptome, tumor, microenvironment

## Abstract

One-carbon (1C) metabolism plays a key role in biological functions linked to the folate cycle. These include nucleotide synthesis; the methylation of DNA, RNA, and proteins in the methionine cycle; and transsulfuration to maintain the redox condition of cancer stem cells in the tumor microenvironment. Recent studies have indicated that small therapeutic compounds affect the mitochondrial folate cycle, epitranscriptome (RNA methylation), and reactive oxygen species reactions in cancer cells. The epitranscriptome controls cellular biochemical reactions, but is also a platform for cell-to-cell interaction and cell transformation. We present an update of recent advances in the study of 1C metabolism related to cancer and demonstrate the areas where further research is needed. We also discuss approaches to therapeutic drug discovery using animal models and propose further steps toward developing precision cancer medicine.

## 1. Introduction

**Figure 1:**
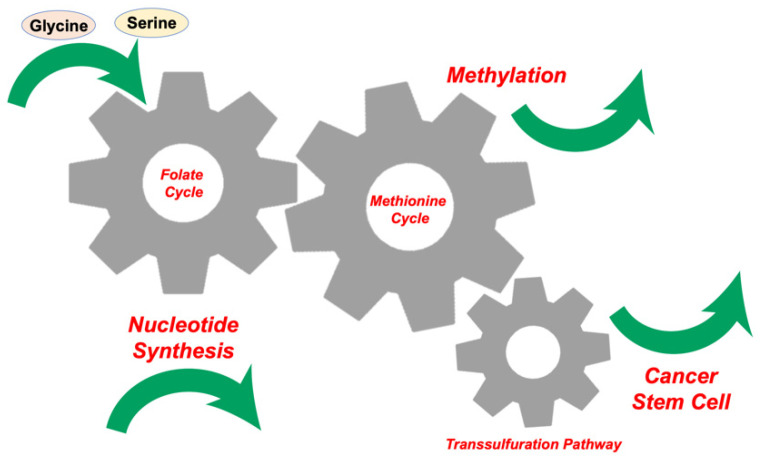
Inputs and outputs of one-carbon (1C) metabolism. The input amino acids, glucose, and metabolites fuel 1C metabolism, which generates various cellular outputs, such as nucleotide synthesis, methylation, and origination of cancer stem cells.

In every cell, physiological processes are controlled by both DNA and prenatally and/or postnatally acquired epigenetic modifications of DNA, RNA, and histone proteins. Epigenetic changes control gene expression and silencing [1]. One-carbon (1C) metabolism includes both the folate and methionine cycles and enables cells to manufacture 1C units (also known as methyl groups), which are used for both methylation reactions and the synthesis of crucial anabolic precursors necessary for life (Figure 1). In the reaction, S-adenosyl methionine (SAM), a product of 1C metabolism, can provide the methylation donors actively [2]. One-carbon metabolism has emerged as a therapeutic target for cancer, which shows unregulated proliferation and uncontrolled cellular differentiation [3]. Our previous studies indicated that serine hydroxymethyltransferase 2 (SHMT2) and 5,10-methylene tetrahydrofolate dehydrogenase 2 (MTHFD2), the first and second enzymes of serine catabolism in mitochondrial 1C metabolism, are independent prognostic factors and therefore potential cancer chemotherapeutic targets [4]. Computational survival rate analysis has indicated that aldehyde dehydrogenase 1 family member L2 (ALDH1L2) is another therapeutic target linked to 1C metabolism [4]. Given that the SHMT2 and MTHFD2 genes are attractive targets for cancer therapy [5], small-molecular inhibitors have been developed to target the folate pathway of 1C metabolism, some of which have been studied in preclinical and clinical trials [6].

SAM in 1C metabolism is involved in the methylation reactions of various downstream cancer therapy targets, including DNA, RNA, and proteins. The downstream targeting of RNA is thought to be a more effective and precise possible therapeutic approach due to the following findings: (1) molecular and imaging studies have revealed an intimate connection between RNA modifications, such as in the epitranscriptome, and in chromatin structure, as shown in [7]; (2) studies of clinical cancer samples have found numerous alterations in the epitranscriptome [8,9]; (3) alterations of the epitranscriptome (RNA modifications) are associated with changes in 1C folate metabolism and increased aerobic glycolysis in cancer cells (the Warburg effect), where cancer cells evade immune surveillance in the tumor microenvironment [10]; and (4) a recent discovery indicating that targeting methyltransferase-like 3 (Mettl3), a key RNA methylation enzyme, resulted in the efficient suppression and eradication of hematopoietic malignancies by a small compound, STM2357 [11]. The RNA methylation reaction can be activated through cooperative binding to the Mettl4–Mettl14–Wtap active binding site by a small molecule [12], suggesting the druggability of this RNA methylation mechanism. These data suggest the importance of the epitranscriptome in cancer [13,14]. In this paper, we present an update of recent advances in the study of cancer 1C metabolism, with a focus on the epitranscriptome. Given that 1C metabolism plays a role in tumors, the targeting may be feasible for precision medicine. We also discuss approaches to therapeutic drug discovery in animal models and propose further steps toward developing precision cancer medicine.

## 2. Glycine, Serine, and Methionine Control 1C Metabolism in the Tumor Microenvironment

**Figure 2:**
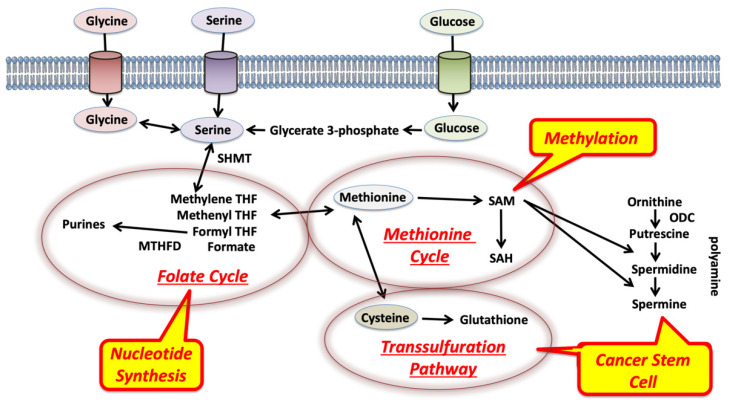
Biochemical schema of 1C metabolism. One-carbon metabolism couples with three reactions: folate cycle, methionine cycle, and transsulfuration pathway [16]. The folate cycle produces purines and nucleotide synthesis. The methionine cycle plays a critical role in the control of methylation of target DNA, RNA, and proteins. The transsulfuration pathway is involved in the regulation of cancer stem cells by the reduction control of cells. Polyamines control cell proliferation and differentiation in cancer stem cells. ODC, ornithine decarboxylase; SAM, S-adenosylmethionine; SAH, S-adenosylhomocysteine.

**Figure 3:**
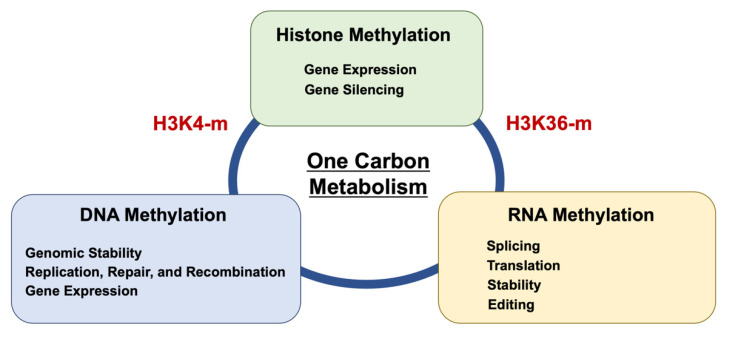
Biochemical linkage and competition of methylation among DNA, RNA, and histone. One-carbon metabolism links the methylation reaction. DNA methylation is mediated by H3K4 methylation [18]. Histone H3 trimethylation at lysine 36 guides m6A RNA modification [19]. Epigenetic methylation of N6-adenine and N6-adenosine with the same input resulted in different output in various biological aspects [20].

Amino acids are a general term for organic compounds containing amines and carboxylic acid functional groups. Although there are more than 500 types of amino acids in nature, only 22 amino acids are present in living organisms [15]. These amino acids exist not only as constituents of proteins that make up various tissues but also in free form in cells and plasma. The amino acids themselves proliferate directly through amino acid transporters [15]. Nine types of amino acids, termed essential amino acids, are not synthesized in the body and are deficient unless ingested from the diet. Among these, serine, glycine, and methionine are involved in the coupling of folate and methionine units in 1C metabolism (Figure 2). Hyperactivation of this pathway is a driver of oncogenesis and establishes a link between epigenetics and cancer [16]. As mentioned above, the functions of 1C metabolism include biosynthesis (purines and thymidine in nucleotides), amino acid homeostasis (glycine, serine, and methionine), epigenetic maintenance (methyl transfer to nucleotides and proteins), and redox defense [17], indicating that 1C metabolism is involved in broad function. Thus, although targeting 1C metabolism is expected to provide new opportunities for translation into cancer precision medicine [3,16], specific targeting will be necessary to control diseases (Figure 3).

Amino acid metabolism is significantly modified in the tumor microenvironment [21]. Alterations in the metabolism of amino acids glycine [17], serine [17], methionine [17], glutamine [22], sarcosine [23], aspartate [24], and cysteine [25] have been previously linked to cancer cell metabolism and malignant phenotypes of various tumors. Recent studies have revealed differential control of amino acids in epithelial cancer cells, mesenchymal cells, and immunocytes, suggesting the potential for amino acids and their transporters, i.e., solute carriers (SLC), in T-cell immunotherapy for cancer [26].

## 3. Competition for Methionine Upstream in 1C Metabolism

**Figure 4:**
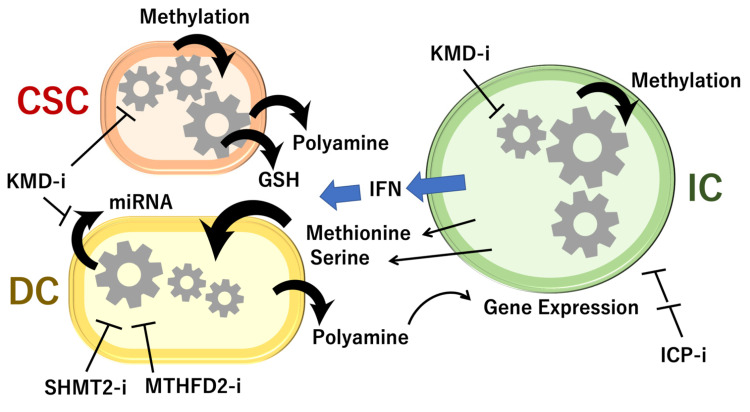
Cell-to-cell interaction of 1C metabolism. GSH, glutathione; SHMT2-i, inhibitor to serine hydroxymethyltransferase 2; MTHFD2-i, inhibitor to mitochondrial bifunctional methylenetetrahydrofolate dehydrogenase/cyclohydrolase; KDM-i, inhibitor to lysine demethylase; IFN, interferon; CSC, cancer stem cell; DC, daughter cancer cell; IC, immune cell. Drug discovery within 1C metabolism pathways may give rise to targets for cancer therapeutics [3,32].

**Figure 5:**
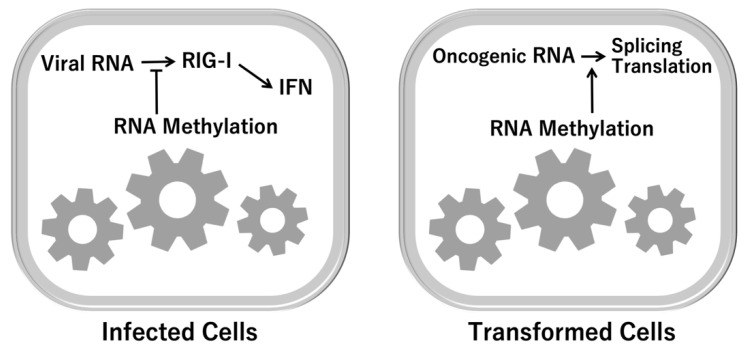
RNA-dependent immune response. As an output of 1C metabolism, RNA methylation is involved in the recognition of and response to exogenous RNA from viruses. RIG-I ATPase assists in the discrimination between self-RNA and non-self-RNA [33,34]. RIG-I, retinoic acid-inducible gene I; IFN, interferon.

Among the three amino acids, i.e., serine, glycine, and methionine, which are catabolized in 1C metabolism, methionine has been implicated in competition between cancer and immune cells, although the mechanism in the remaining two amino acids, serine and glycine, is unclear. Cancer cells compete with each other or with immune cells for methionine and thus impair CD8 T-cell function in vivo [27]. This occurs through interference with 1C metabolism, resulting in altered histone methylation [28] but also in methylation of nucleotides (Figure 4 and Figure 5). The same study found that the targeting and inhibition of the methionine transporter SLC43A2, which is highly expressed in tumor cells, resulted in the restoration of H3K79me2 histone modification in T cells, which led to higher checkpoint-induced tumor immunity [28]. Cancer cell methionine consumption is a critical mechanism that fuels cancer cells but also impairs anti-tumor immunity (Figure 4 and Figure 5) [28].

Although the RNA modification is involved in the acquired immune recognition of antigens of exogenous nucleotides, such as viral or bacterial infections, recent studies indicated that innate dendritic cells exposed to such modified RNAs ablated the activity [29]. Given that the innate dendritic cells and Toll-like receptors (TLRs)-expressing cells were potently activated by bacterial RNA and mitochondrial RNA, but not by mammalian total RNA, it is proposed that the RNA modifications suppress the activity of dendritic cells, and the innate immune system may detect modification-null RNAs from bacteria [29]. Furthermore, a recent study indicated that m6A RNA modification on human circular RNAs (circRNAs), a prevalent form of foreign nucleotides, inhibits innate immunity [30]. The study of modification-null circRNA demonstrated that they directly activate RNA pattern recognition receptor RIG-I to induce the filamentation of the adaptor protein MAVS and activation of the downstream transcription factor, interferon regulatory factor 3 (IRF3), which can contribute to the targeting and eradication of exogenous RNAs [30]. However, positions and patterns of RNA modification to elicit immunity remains to be understood perfectly. Interestingly, a recent study of methylated RNA immunoprecipitation sequencing (MeRIP-seq) and RNA transcriptome sequencing (RNA-seq) showed that AlkB homolog 5 (ALKBH5)-dependent high-mobility group box 1 (HMGB1) expression mediates the stimulator of interferon genes protein (STING)-IRF3 innate immune response in radiation-induced liver diseases, as unavoidable liver injury, which is the adverse effect for treatment of primary liver cancer [31]. Irradiation induced the recruitment of ALKBH5 to demethylation sites of m6A residues in the 3′ untranslated region (UTR) of HMGB1, which resulted in activation of STING-IRF3 signaling [31]. The study suggested that the RNA modification at positions in the UTR of HMGB1 gene is involved in the innate immune response in the radiation-induced liver damage in cancer treatment. Taken together, the RNA modification plays a role in the function of innate and acquired immunity.

Methionine plays a critical role in 1C metabolism and controls the methionine cycle, which couples with the folate cycle and transsulfuration pathway. Cancer stem cells survive after chemotherapy and radiation therapy [35], in which several cell-surface markers are involved, such as CD133 [36], c-Met [37], and CD44 [38]. Generally, it is considered that cancer stem cells survive in the low-stress condition after exposure to chemotherapy and radiation therapy through the mechanism of slow-cycling characteristics [39], activation of glycolysis and the pentose phosphate pathway [40], and low levels of glutathione [41]. The 1C metabolism couples with polyamine metabolism, which is involved in the production of SAM, in the pathway of ornithine, putrescine, spermidine, and spermine [42]. The SAM provides the methylation donor to DNA and RNA. The demethylation reaction of methylated forms of the substances can be mediated in the nucleus via enzymes, such as iron-dependent dioxygenases [43]. Among the demethylation enzymes, lysine demethylase (KDM) 5 plays a role in slow-cycling cancer stem cells, which are required for continuous cancer growth [44]. The depletion of KDM resulted in the induction of cellular senescence in gastrointestinal cancer [45]. In the proliferating daughter cancer cells, the inhibition of 1C enzymes in mitochondria, Shmt2 and Mthfd2, is supposed to induce tumor eradication [32]. In the tumor microenvironment, immune cells and cancer cells compete for methionine, which is involved in the production of anti-tumor cytokines, such as interferon (Figure 4). Thus, the hypothesis is proposed that the 1C metabolism plays an important role in the initiation of cancer in the early stages, maintaining cancer stem cells, advancement of cellular transformation, and expression of malignant behaviors of tumors, in the tissue microenvironment [46]. Studies of 1C mechanism will provide candidates for drug discovery.

Recent studies indicated that methylation of RNA is involved in the therapy resistance and proliferation of pancreatic cancer cells, via reaction of RNA demethylation enzyme Mettl3 [47]. The expression of RNA methylation and responses are under the control of oncogene c-MYC [48]. In addition to cancer development and cellular transformation, the methylation of RNA is involved in the recognition of endogenous RNA or exogenous viral RNA (Figure 5), suggesting that the biological response system of methylation occurs as a result of 1C metabolism.

Glycine, the smallest nonessential amino acid, is involved in various aspects of cancer, and a previous study of metabolic profiling showed that glycine plays a critical role in the rapid proliferation of cancer cells [49]. Glycine participates in the synthesis of proteins and glutathione as well as in detoxification reactions, which are involved in a broad spectrum of anti-inflammatory, cytoprotective, and immunomodulatory properties, all elements critical in the cancer microenvironment [50].

Serine metabolism is also frequently dysregulated and highly expressed in cancer cells. Serine synthesis facilitates 1C-related amino acid transport, nucleotide synthesis, folate metabolism, and redox homeostasis—all mechanisms involved in the development of cancer [51]. Sideroflexin 1 (SFXN1), a serine transporter and multipass inner mitochondrial membrane protein, was recently discovered by a CRISPR-based genetic screen in human cells [52]. Although any involvement of SFXN1 in the development of cancer is unclear, it has been linked to gene expression associated with osteoarthritic synovitis [53].

## 4. Epitranscriptome (RNA Methylation)

Recent studies revealed the function of RNA modifications through the mechanisms of control of splicing, stability of transcripts, and translation process of encoded peptides [14]. Given that m6A, i.e., N6-methyl adenosine, is a predominant modification of RNA, previous studies demonstrated that the methylation reaction occurs according to consensus sequence, typically GGACU; more precisely, RRm6ACH (R notes G or A; H includes A, C, or U), or Pu (G > A) m6AC (A/C/U) (where Pu represents purine), though there are some exceptions (reviewed in [14]). The RNA modification is regulated finely by the methylation “writing” enzymes, as a forward reaction, such as Mettl3, which contains a catalytic domain, in the protein complex with Mettl14 and Wilms’ tumor 1-associating protein (Wtap) as subunits [14]. Mettl3 forms a heterodimer with Mettl14 to induce methylation of adenosine at the N6 position of the RNA. Additionally, Mettl3 constitutes the catalytic site in the heterodimer formed with Mettl14. Accordingly, m6A in the 5′-[A/G]GAC-3′ consensus sequence of mRNA plays a role in the function, such as stability, processing, translation efficiency, and editing [14]. Considering that m6A modification of RNA is involved in the circadian clock, differentiation of embryonic stem cells and hematopoietic cell lineages, cortical neurogenesis, DNA damage response, T-cell homeostasis and differentiation, and primary miRNA processing, the epitranscriptome plays a role in the heterogeneity of tumors [14]. Eventually, m6A is required for T-cell homeostasis and differentiation. In mice, the naive Mettl3-deficient T cells inhibit the mRNA decay and increase the levels of suppressor of cytokine signaling (SOCS) family members, which inhibit the signaling of a transcription factor, signal transducers and activators of transcription (STAT) [54]; this mechanism consequently inhibited IL-7-mediated STAT5 activation and T-cell homeostatic proliferation and differentiation, suggesting the biological role of m6A modification in T-cell-mediated pathogenesis and T-cell homeostasis through the mechanism of signal-dependent induction of mRNA degradation [54].

In the reverse de-methylation reactions, “erasing” enzymes are involved, including fat mass and obesity-associated protein (FTO) and α-ketoglutarate-dependent dioxygenase (AlkB) homolog 5 (ALKBH5) [14]. The genes or proteins were identified through the characteristics of obesity-associated phenotypes, suggesting metabolism is involved closely in the control of the epitranscriptome in the cells. Given that the 1C metabolism provides the methylation donor to target molecules, and amino acids such as extracellular methionine, serine, and glycine can fuel cancer cells and immune T cells in the tumor microenvironment, it is proposed that those amino acids play a critical role upstream of 1C metabolism, and thus to eradicate the malignant cancer stem cells and daughter cells, the targeting of the 1C metabolism pathway stands to reason. Moreover, the function of RNA modification can be exerted via “reading” enzymes, such as the protein family of heterogeneous nuclear ribonucleoproteins (hnRNP) and YT521-B homology (YTH) m6A RNA binding protein 1 (YTHDF1). Those reading enzymes recognize the methylation of RNAs and bind to different proteins, which finally affect the translation efficiency and lifetime of RNA [14]. Given that a recent study demonstrated that the oncogene c-MYC promotes the expression of YTH domain family protein genes [32], it is necessary to develop adequate biomarkers, or surrogate markers, which can prove the relationship with the final evaluation of diagnostic and therapeutic behavior and drug efficacy, in medical and pharmaceutical research. Given that 1C metabolism places the methyl residue as a “fingerprint” on the target molecules, the amounts and sites of m6A may associate with the intracellular 1C metabolism and the measurement of the methylation status is proposed to be useful for monitoring diseases as biomarkers. The recent study showed that the measurement of RNA methylation is more sensitive and specific for the prediction of early stages of gastrointestinal cancer including pancreatic cancer, compared to those of the expression amount of RNA [8]. Taken together, the measurement of the epitranscriptome will be useful for monitoring 1C metabolism.

Considering it is important to maximize anti-cancer effects and to minimize adverse events, the novel therapeutic approaches will also need increasing specificity and pharmacological effects. The recent study indicates that the SLC43A2 methionine transporter is a candidate for the target with high specificity in the tumor microenvironment [28]. Not only small compounds, but also therapeutic strategies such as nucleotide medicines, antisense, shRNA, and genome editing, may be plausible for pre-clinical trials.

## 5. The Significance of RNA Modification

We can suggest the significance and implication of RNA control via m6A modification, in comparison with the expression control of each gene promoter or post-translational levels, as the following. Firstly, the “erasing” enzymes are related closely to metabolic diseases, such as the “Fat Mass and Obesity Related” (FTO) gene, the mechanisms of which can impact on obesity and energy balance (reviewed in [55]). The previous studies indicated that the FTO gene is widely expressed in the brain, including hypothalamic nuclei linked to food intake regulation in rodents, and the activity that the gene product plays a role in is associated with an amino acid sensor, linking circulating amino acids to the mammalian target of rapamycin complex 1 (mTORC1) [55]. Accordingly, the reaction of RNA modification is supposed to reflect the condition of diet and obesity in bodies. Accordingly, RNA modification has emerged as a potential target of human diseases, such as cancer [56]. Second, the intracellular dynamic process is involved in RNA modification. RNA modification plays a role in splicing at the post-transcriptional level, nuclear exportation, and translation in the cytoplasm. The de-methylation is mediated by the nuclear enzymes, which can reflect the nuclear condition, such as open and closed, of chromatin in each cell. Recent studies have placed emphasis on the elucidation of the vast heterogeneity and context-dependent functions of RNA methylation “writers”, “readers”, and “erasers”, which are eventually complicated by divergent cell-type-specific and tissue-specific expression and localization of the effectors, as well as modifications, with abundant expression of different RNA species (reviewed in [57]). Third, RNA modification responds to extracellular stimuli. Hypoxia and alterations of nutrients such as glucose and amino acids, growth factors such as epidermal growth factors and transforming growth factor, and cytokines signaling are involved in the function of iron-dependent dioxygenases [57]. The response can be executed in a relatively prompt manner to the extracellular condition by post-transcriptional modification, as with the pre-translational process. The diversity of RNA modification was observed in microRNAs in non-coding RNAs, in extracellular vesicles (exosomes) [8]. The diversity of RNA modification can contribute to the heterogeneous characters of tumors [14]. Fourth, RNA modification affects its function through RNA editing, a unique mechanism which can alter the cellular fate of RNA molecules but also changes their sequence relevant to the genome, as noted in several cases in human diseases [58], suggesting that it is not an exaggeration to say that the effect of the RNA modification is in a sense beyond the classical central dogma of molecular biology, i.e., an explanation of the flow of genetic information within a biological system. Finally, recent studies emerged that RNA modification has been found to play an essential role in the regulation of the immune system [59]. RNA modification plays a role in various aspects of immunity, including immune recognition of antigens and target cells, and activation of innate and adaptive immune responses in epithelium, mesenchyme, and lymphoid organs, which can contribute to cell fate decisions and immune function [59]. Accordingly, it is suggested that RNA modification may be useful for the biomarkers to assess immune reaction in human diseases. Taken together, RNA modification is expected to provide novel seeds for the diagnosis and therapy of gastrointestinal cancer, which reflect the critical one-carbon metabolism.

## 6. Animal Models Reveal Cell-to-Cell Interactions of 1C Metabolites

MicroRNAs, types of small non-coding RNAs of around 22 bp in length, were discovered as regulators of developmental timing in the nematode *Caenorhabditis elegans* [60,61]. MicroRNAs have also been shown to play an important role in determining both cell fate and cell identity in *C. elegans* [62,63]. MicroRNAs may be used as a mechanism of RNA interference [64], but also in the development of therapeutic targets of human diseases including tumors [65,66]. The latest version of the public repository miRbase contains annotations for 48,860 mature microRNAs across 271 organisms (http://www.mirbase.org accessed on 1 January 2019).

*C. elegans* is an appropriate animal model for the study of altered 1C folate metabolism as most enzymes show a high degree of similarity to their human counterparts, especially the 64% involved with total methionine synthase. Impaired folate metabolism in humans can increase birth defects, neurodegenerative disorders, cardiovascular diseases, and cancer [67]. Previous work on RNA interference showed that the methionine synthase (MS) and thymidylate synthase cycles are involved in adaptation mechanisms during folate deficiency and over-supplementation [67]. Furthermore, the biguanide drug metformin, which is widely used for type 2 diabetes, caused an increased lifespan in *C. elegans* cocultured with *Escherichia coli* as a feed for the nematodes [68]. Metformin-induced longevity was linked to mutations of MS and SAM synthase in the worms, suggesting that metformin acts as a dietary restriction mimetic [68]. Interestingly, the previous study also indicated that metformin-related changes in worm lifespan depended on the *E. coli* strain’s metformin sensitivity and environmental glucose concentration [63]. This finding implicates the gastrointestinal organs of higher vertebrates through a complex mechanism, such as inflammation [69].

**Figure 6:**
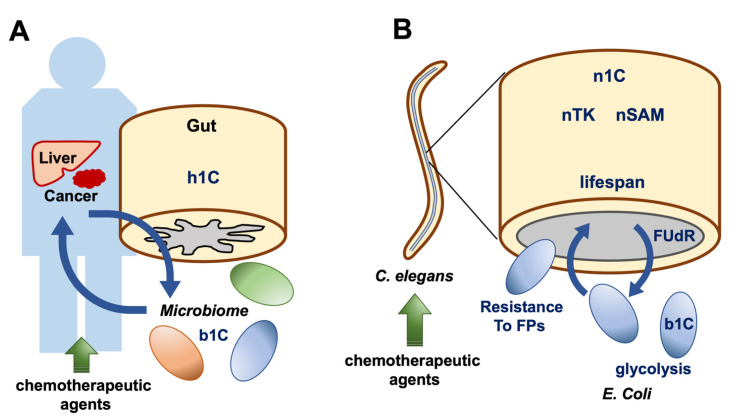
(**A**) Human gut in a patient with cancer: h1C, human one-carbon metabolism; b1C, bacterial one-carbon metabolism. (**B**) An animal model using *Caenorhabditis elegans* to study 1C metabolism in the microbiome. In the intestines of nematodes, bacteria produce metabolites from b1C, which in turn fuel the host nematodes. Exposure to chemotherapeutic agents resulted in 1C alterations in nematodes and bacteria. Analysis of this model may give insights into mechanisms of microbiome-to-host interaction in humans. *C. elegans*, *Caenorhabditis elegans*; n1C, nematode one-carbon metabolism; nTK, nematode tyrosine kinase; nSAM, nematode S-adenosyl methionine; FUdR, 5-fluoro 2′-deoxyuridine; FPs, fluoropyrimidines; *E. coli*, *Escherichia coli*.

Another recent study revealed that bacterial metabolism in the *C. elegans* gut can affect the nematode’s reaction to anticancer agents [70]. A further study of *C. elegans* indicated that diet–microbe interactions can alter the host response to chemotherapeutic reagents such as 5-fluoro 2′-deoxyuridine (FUdR) [66]. This study showed that dietary serine does not alter FUdR metabolism, but it changes *E. coli*’s 1C metabolism, which exacerbates host drug toxicity [71]. On the other hand, the evolved resistance of *E. coli* against fluoropyrimidines can exert other effects, such as a reduction in the impact of chemotherapies on *C. elegans* [72]. These findings suggest that the model *C. elegans* is useful for the elucidation of metabolic interactions between host and microbiome or between epithelial cancer and mesenchymal differentiated cells (Figure 6).

## 7. RNA Methylation in *Caenorhabditis elegans*

Studies of transfer (t)RNA methylation in *C. elegans* indicated that 5-methyl cytosine (m5C) loss leads to reduced translation efficiency of UUG-rich transcripts and impaired fertility, suggesting a role for m5C tRNA methylation in translational adaptation to higher temperatures or heat stress [73]. Another recent study of ribosomal (r)RNA in *C. elegans* indicated that the loss of a single enzyme, NOL1/NOP2/Sun Domain Family, Member 1 (NSUN-1), which is required for rRNA methylation, has highly specific effects on the organism’s development and physiology [74]. The methylation of miRNAs in *C. elegans* remains unclear.

## 8. Conclusions

Although cancer is a genetic disease with various DNA sequence alterations, recent studies have demonstrated that epigenetic changes, especially of the epitranscriptome, play a critical role in tumor development, mediated by 1C metabolism. Targeting 1C metabolism has the potential to inhibit malignant tumor behaviors, as shown by the effects of drugs targeting Mthfd2, Shmt2, and Mettl3. To assess the effect and underlying mechanisms of potential therapies in preclinical studies, animal models, such as *C. elegans*, will be useful to guide researchers toward precision cancer medicine.

## Data Availability

N.A. on this review article.
